# Clinical Evidence for the Benefits of Burosumab Therapy for X-Linked Hypophosphatemia (XLH) and Other Conditions in Adults and Children

**DOI:** 10.3389/fendo.2020.00338

**Published:** 2020-05-28

**Authors:** Aaron Schindeler, Andrew Biggin, Craig F. Munns

**Affiliations:** ^1^Bioengineering and Molecular Medicine Laboratory, The Children's Hospital at Westmead, Westmead, NSW, Australia; ^2^Discipline of Child and Adolescent Health, University of Sydney, Sydney, NSW, Australia; ^3^Department of Endocrinology and Diabetes, The Children's Hospital at Westmead, Westmead, NSW, Australia

**Keywords:** burosumab FGF-23, XLH, X-linked hypophosphatemic rickets, KRN23, treatment, review

## Abstract

Burosumab (KRN23) is an FGF23 neutralizing antibody that has been the subject of several recent clinical trials principally focused on the treatment of hypophosphatemic rickets in patients with X-linked hypophosphatemia (XLH). Since the first publications in 2014, these trials have demonstrated efficacy with minimal safety concerns in both adult and pediatric cohorts. These studies have used dose-escalation to establish a dosing regimen that is well-tolerated in clinical use. This review summarizes the clinical trial data with respect to burosumab treatment in adults and children as well as noting several clinical trials currently underway. While burosumab appears transformative for the treatment of XLH, long term follow-up studies would be required to allay concerns over the potential for nephrocalcinosis and cardiac calcification. While these do not appear to be problematic in current trials, the effects of chronic or lifelong treatment have yet to be established.

## Introduction

Fibroblast growth factors (FGFs) are a family of proteins important for the regulation of a range of physiological processes (1). FGF23 came to light almost two decades ago as a molecular cause of autosomal dominant hypophosphatemic rickets (2). Since then, the regulation of serum phosphate has been revealed to involve a complex crosstalk between FGF23 and a FGFR co-receptor Klotho that works in concert with signaling by the PTH/1,25(OH)_2_D axis (3). This signaling involves multiple organ/tissue systems including the bone compartment, kidneys, digestive system, and the parathyroid gland.

X-linked hypophosphatemic rickets (XLH) is a genetic disorder associated with mutations in the *Phosphate Regulating Endopeptidase Homolog X-Linked (PHEX)* gene. Inactivating mutations in *PHEX* result in an upregulation of FGF23 expression that leads to hypophosphatemia through reduced tubular reabsorption of phosphate and down regulation of 1α-hydroxylase activity (4). The dysregulation of phosphate homeostasis in XLH cannot be normalized with cholecalciferol (in contrast to nutritional rickets) and leads to abnormal bone development and short stature. Individuals with XLH also suffer from bone and muscle pain, impaired ambulation, and an elevated risk of dental complications. Osteomalacia can also lead to bone deformities that need to be managed by orthopedic intervention. Nevertheless, the symptomology of XLH varies in severity and onset between individuals.

## Conventional Therapy for XLH

Conventional management of XLH involves phosphate supplementation in an attempt to provide adequate phosphate to allow for bone mineralization and normal differentiation of growth plate chondrocytes. In parallel with phosphate supplements, active vitamin D is normalized by supplements with calcitriol (1,25 dihydroxy vitamin D; 1,25(OH)_2_D) or alfacalcidol (5). Active vitamin D is also administered to offset the hypocalcemic effect of phosphate supplementation and prevent the development of hyperparathyroidism. Unlike nutritional rickets, cholecalciferol therapy alone is insufficient for the treatment of hypophosphatemic rickets. Despite this, many physicians ensure 25 hydroxy vitamin D (25OHD) levels are kept within the sufficient range to meet the potential “off-bone” effects of 25OHD.

The review by Linglart et al. (5) describes in detail the conventional treatment of hypophosphatemic rickets by supplementation as well as pharmacological and non-pharmacological management of symptoms. There remains a major challenge around dental and periodontal complications, and a proactive approach to oral health is recommended. While conventional therapy can help manage bone pain, children with XLH can still develop significant lower limb deformity and often have short stature. Human growth hormone (hGH) has been clinically used as an adjunctive therapy to increase height, data from published reports showed poor efficacy (6, 7). Moreover, such conventional approaches do not deal with the underlying dysregulation of phosphate homeostasis.

## Adult Clinical Trials for Treating XLH With Burosumab

Burosumab (KRN23) is a neutralizing antibody to FGF23 that has emerged as a promising treatment for XLH and hypophosphatemic rickets. This arose from work using a hypophosphatemic mouse model (*Hyp* mouse) that models XLH where neutralizing FGF23 antibodies were found to rescue the phenotype (8). Since then burosumab has undergone a number of clinical trials that have produced significant clinical outcomes in patients. This review summarizes the results of all currently published clinical trials and discusses the future potential for burosumab in treating other conditions associated with dysregulated phosphate homeostasis.

All of the initial trials for burosumab to examine safety and efficacy were performed in adults with XLH. These are summarized in Table 1, although in some cases multiple papers report data from a single trial cohort. The first published trial for burosumab in 2014 was a double-blind placebo-controlled trial that compared single (escalating) doses of drug given subcutaneously or intravenously (9). It was designed to investigate the pharmacokinetics, pharmacodynamics, immunogenicity, safety and tolerability of burosumab over a 50 day period. The cohort was comprised of adults with a diagnosis of XLH screened against a range of exclusion criteria devised to avoid confounding factors. No patient was given Vitamin D, calcium or phosphate supplements from 10 days prior to burosumab treatment through to the end of the study. Nausea (24%) and headache (18%) were the most commonly reported side-effects, but none that were deemed to be serious or led to withdrawal from the study. The original paper specifically noted that there were no incidents of nephrocalcinosis or situations resulting in hypercalciuria, hypercalcemia, or biochemical markers that would lead to clinical concern. Subsequent studies were also published that further explored the pharmacokinetics and pharmacodynamics of burosumab in this patient cohort (11, 12).

**Table 1:** Adult clinical trials involving burosumab for XLH.

**References**	**Cohort**	**Phase**	**Study design**	**Burosumab dose/administration**	**Outcomes**
Carpenter et al. (9)	38 adults with XLH	I	Double-blind placebo-controlled randomized trial	0.003–0.3 mg/kg i.v. or 0.1–1 mg/kg s.c. single dose (vs. placebo)	Therapy increased TmP/GFR, serum Pi, and serum 1,25(OH)2D. Calculated mean t1/2 after i.v. vs. s.c. administration.
Imel et al. (10)	28 adult with XLH; 22 in 12 months extension study	I/II	Open-label trial (dose escalation study)	0.05 mg/kg, 0.1 mg/kg, 0.3 mg/kg, 0.6 mg/kg s.c. escalating every 4 w	Therapy increased TmP/GFR, serum Pi, and 1,25(OH)2D.
Zhang et al. (11)			Cohorts from Carpenter et al. (9) and Imel et al. (10)		Described PK of burosumab using 1-compartment model.
Zhang et al. (12)			Cohort from Imel et al. (10)		Described mean time to reach maximum serum burosumab levels and half-life.
Carpenter et al. (13)	16 patients with TIO or ENS	II	Open-label trial (single-arm dose finding study)	0.3–2.0 mg/kg s.c. given every 4 w	Therapy increased TmP/GFR, serum Pi, and serum 1,25(OH)2D. One serious adverse event (neoplasm progression).
Ruppe et al. (14)			Cohort from Imel et al. (10)		Validated alternative quality of life questionnaires in treated XLH individuals.
Insogna et al. (15)	134 adults with XLH (age 18–65), confirmed *PHEX* mutation, and meeting other criteria	III	Double-blind placebo-controlled randomized trial (24 w primary analysis)	1 mg/kg s.c. given every 4 w (vs. placebo)	Therapy improved WOMAC stiffness subscale but not some other measures. Acceptable safety profile.
Portale et al. (16)	Cohort from Insogna et al. (15)	III	Extension (Insogna study)—open-label period	1 mg/kg s.c. given every 4 w	Therapy enabled maintenance of normal serum Pi and there was an increased in healed fractures. Physical outcome measures were improved by therapy.
Insogna et al. (17)	11 patients with paired biopsies.	III	Double-blind placebo-controlled randomized trial	1 mg/kg s.c. given every 4 w (vs. placebo)	Therapy improved osteomalacia as measured by bone histomorphometry.
Cheong et al. (18)	15 Japanese/Korean XLH patients (*n* = 5 per cohort)	I	Open-label (dose escalation study)	0.3 mg/kg vs. 0.6 mg/kg vs. 1.0 mg/kg s.c. single dose	Therapy increased TmP/GFR, serum Pi, and serum 1,25(OH)2D with no serious adverse events.

An open-label dose escalation study by Imel et al. (10) used multiple monthly doses as well as a higher maximal dose (0.6 mg/kg s.c.) than previously reported by Carpenter et al. (9). The study did not include a control group, but did feature a 12-month extension study entered by 76% of participants. Compliance was high and the primary outcome of serum Pi increased in response to burosumab, although it did decrease toward the end of the monthly dosing cycle. Secondary measures such as TmP/GFR, 1,25(OH)_2_D and bone markers also showed positive responses to treatment. The overall adverse event (AE) rate was extremely high, however many of these were of mild-moderate severity (including an elevated rate of restless leg syndrome) or not specifically linked to the drug treatment. Drug-related AEs included diarrhea and arthralgia and injection site reactions. Six major AEs occurred, but they were all deemed to be unrelated to the drug. Notably, patients underwent safety monitoring including cardiac CT and renal ultrasonograms, with one individual showing increases in coronary calcification and no subjects showed evidence of progressive nephrocalcinosis.

Following the results of these studies, a number of open-label trials for burosumab have been published and showed comparable findings. Ruppe et al. (14) reported on the cohort of Imel et al. (10), the focus of this latter study being on quality of life outcomes. In addition to showing positive clinical outcomes similar to those previously reported, this report described the validation of the SF-36v2 Health Survey (SF-36v2) and the Western Ontario and McMaster Osteoarthritis Index (WOMAC) questionnaires to measure quality of life and its change in response to treatment. The WOMAC measures of physical functioning and stiffness were both found to be significantly improved by burosumab (14). A first-in-Asian trial showed similar biochemical responses with no signal for increased risk in a Japanese/Korean cohort (18).

A large international multicenter trial first described by Insogna et al. reported on 134 adults recruited with stricter selection criteria than prior studies including a confirmed *PHEX* mutation (15). This double-blind, placebo-controlled, randomized trial ran for 24 weeks and was followed by an open label extension (16). Of note, the control group did not receive phosphate or calcitriol. The initial publication indicated no baseline differences between the treatment and placebo cohorts, but those treated with burosumab had a considerable improvement in serum phosphate (15). The burosumab group showed 94% above the lower limit of normal (LLN) across the midpoints of monthly dosing compared to 7% in the placebo group. The burosumab group also exhibited an improvement in the key secondary endpoints including pain, WOMAC physical function and fracture healing; strongly supporting the clinical efficacy of burosumab in adult XLH treatment. No significant changes from baseline were observed for the 6-min walk test. Again, while the rate of adverse events was high, issues such as injection site reactions were similar between groups. No serious AEs were considered to be related to study treatment. This was followed by reports illustrating the positive effects on bone histology performed on bone biopsies taken from treated patients with *PHEX* mutations (17). Thirteen adults underwent histomorphometric analysis of transiliac bone biopsies following burosumab treatment. All osteomalacia-related measures (including osteoid volume/bone volume, osteoid thickness, osteoid surface/bone surface, and mineralization lag time) showed significant improvement following 48 weeks of treatment. The open label extension showed continuing benefits to patients over a further 24 weeks including those on the placebo arm that received burosumab in the extension. While nephrocalcinosis scores did not considerably change, it must be acknowledged that the long-term effects of years of burosumab therapy remain unknown.

## Pediatric Clinical Trials for Treating XLH With Burosumab

Since 2018, four studies have published clinical data on the efficacy and safety of burosumab therapy in children with XLH (Table 2). The findings have been broadly supportive regarding the safety and efficacy of burosumab in children.

**Table 2:** Pediatric clinical trials involving burosumab for XLH.

**References**	**Cohort**	**Phase**	**Study design**	**Burosumab dose/administration**	**Outcomes**
Carpenter et al. (19)	52 pediatric XLH patients	II	Open-label trial (dose frequency study)	Dose adjusted to achieve normal serum Pi, given every 2 or 4 w; open label extension option	Therapy increased renal tubular phosphate reabsorption, serum Pi, linear growth, and physical function. Therapy reduced pain and the severity of rickets.
Whyte et al. (20)	13 children with XLH (age 1–4)	II	Open-label multicenter trial (with extension)	0.8 mg/kg s.c. given every 2 w (escalating to 1.2 mg/kg if poor response)	Adverse events but no severe adverse events related to treatment. Therapy decreased rickets severity and may improve growth.
Imel et al. (21)	61 children with XLH (age 1–12), exposed to conventional therapy	III	Randomized trial vs. conventional therapy	0.8 mg/kg s.c. given every 2 w (vs. conventional therapy)	Burosumab therapy gave greater clinical improvements in rickets severity, growth, and biochemistry vs. conventional therapy.
Martin Ramos et al. (22)	5 children with XLH (ages 6–16)	II	Case series	0.8 mg/kg s.c. given every 2 w	1 year treatment showed burosumab to be well-tolerated and showed growth improvement and no hyperphosphatemia.

Carpenter et al. carried out an open-label phase 2 trial in 52 children with XLH and compared 2 and 4 week dosing schedules (19). The primary outcome measure was the Thacher rickets severity score, a measure that assesses radiographs of both knees and wrists. Serum Pi, serum alkaline phosphatase, and other biochemical assays, as well as and functional measures of physical ability were also taken. Adverse events and serious adverse events were also recorded. Both groups recorded a decrease in rickets severity by Thacher score and while the 2 week group showed a lower mean than the 4 week group, this was not reported to be statistically significant. Similarly, biochemical measures were normalized by burosumab, however serum Pi fluctuated considerably less with 2 week dosing compared to every 4 weeks. A range of minor adverse events were reported, particularly injection-site reactions, headaches and cough, although these were less common in the 4 week dosing group. One serious adverse event was reported—one patient was hospitalized for myalgia and fever that were of moderate severity and potentially linked to treatment. This patient continued with treatment and no AEs reoccurred. Following this study, all subsequent pediatric studies have used a burosumab regimen of 0.8 mg/kg every 2 weeks with the possibility of titrating up to 1.2 mg/kg every 2 weeks.

A second trial by Whyte et al. (2019) was an open-label phase 2 trial at three US-based hospital sites, and similarly assessed radiographic, biochemistry, and safety outcomes in toddlers aged 1–4 (20). This trial examined a smaller cohort (13 children with XLH), all completed the 64 weeks of treatment, and there was an optional 96 week extension period. This study similarly reported rapid normalization of serum Pi and improvement in Thacher rickets severity score at 40 and 64 weeks. Mild to moderate adverse events were reported for all patients, such as cough, pyrexia, and respiratory tract infections, and would commonly be seen in over a similar duration in children of this age group. Two cases of injection site erythema (15%) were reported, which are likely related to treatment. One severe adverse reaction (a food allergic reaction) was reported but unrelated to burosumab therapy.

A final recent report was published by a Spanish group by Martin Ramos et al. (22). This group followed a similar protocol to prior groups and described a case series of five children with confirmed PHEX mutations (aged 6–16) who received burosumab therapy. Burosumab was reported to show a positive effect on growth in 3/5 children, where a marked improvement was noted. Families reported that eliminating the need for routine oral phosphate supplementation was seen as a major benefit.

As part of an international multicenter trial, Imel et al. reported on a direct comparison between burosumab and conventional therapy (calcitriol and phosphate) in children with XLH (21). This was an open-label phase 3 trial that recruited 61 children aged 1–12 and lasted for 64 weeks, although the primary outcome was at 40 weeks. Substantial and considerable improvements were seen in Radiographic Global Impression of Change score and Thacher rickets severity score at 40 weeks and sustained at 64 weeks. Biochemical measures such as alkaline phosphatase and serum Pi were also rapidly normalized. Children treated with burosumab also showed a statistically significant improvement in height compared to the conventional therapy group. The burosumab group also showed a significantly greater improvement in distance walked during a 6-min walk test over the same treatment period. Burosumab was well-tolerated, however non-serious adverse events were higher overall compared to conventional therapy. Three serious adverse events were reported in both groups, but were deemed to be unrelated to treatment. Due to the limited accessibility and costs associated with burosumab therapy, this seminal study is the first to provide a direct comparison with traditional management practices. Although burosumab was shown to be clinically superior to conventional therapy, there is yet to be a cost benefit analysis performed. Such data may be required before burosumab is made available within some health care services.

To date, the most comprehensive guide covering the clinical use of burosumab in both adults and children is presented in the 2019 Consensus Statement published in *Nature Reviews Nephrology* (23). A brief summary of the key recommendations for pediatric burosumab therapy are as follows, although these are more detailed by Haffner et al. (23).

- Consideration of burosumab is highly recommended in children with XLH >1 year and in adolescents where overt bone disease is being refractory to conventional treatment and/or conventional treatment is causing complications and/or the patient is unable to adhere to the conventional treatment schedule.
- In children, a starting burosumab dose of 0.4 mg/kg body weight (subcutaneously) given every 2 weeks is recommended, and this can be titrated in 0.4 mg/kg increments to raise fasting serum Pi levels to above the lower range of normal. Hyperphosphatemia should be monitored by regular measurement of fasting serum Pi levels. The dosing should be discontinued if serum Pi is above the upper range of normal. Burosumab dose should not be adjusted more than once every 4 weeks.
- Burosumab is not recommended to be given in conjunction with conventional treatment, or initiated when fasting serum Pi is within the normal reference range, or where severe renal impairment exists.

## Current and Future Burosumab Trials

Burosumab may have some utility outside the treatment of XLH and hypophosphatemic rickets. In 2016 Carpenter et al. presented an abstract describing an unpublished open-label single-arm phase 2 trial for burosumab in adult patients with tumor-induced osteomalacia TIO (*n* = 8) and epidermal nevus syndromes ENS (*n* = 1) (13). TIO is a rare syndrome characterized by bone pain, fractures and muscle fatigue/weakness caused by small mesenchymal tumors that may be found in bone or soft tissue that secrete FGF23. ENS are a group of rare disorders characterized by various extra-cutaneous abnormalities with skin lesions that can also involve hypophosphatemic rickets and elevated FGF23 (24). Burosumab improved serum phosphorous and bone mineralization based on bone biopsies in both patient groups. AEs were considered to be mild and overall burosumab was considered to be potentially beneficial for both TIO and ENS.

Looking ahead to the future of burosumab research, a number of notable trials have been registered on clinicaltrials.gov. It is anticipated that many of the published studies may provide subsequent analysis and follow-up of XLH patients (pediatric and adult) on burosumab to compare long-term benefits with any risks associated with chronic use. Additionally, several other clinical trials have been registered and seek to address other specific questions.

NCT04188964 is a phase 1/2 open-label multicenter trial aimed to study the efficacy, tolerability and safety in infants with XLH under 1 year of age. It is estimated to run from 2020 to 2023 and those recruited will be treated every 2 weeks with 0.4 mg/kg s.c. burosumab for 64 weeks. This will give an insight into the potential for early intervention and elucidate the long-term impact on skeletal development and quality of life.

NCT04146935, estimated to complete in 2021, is examining the effects of burosumab on muscle function. Individuals with XLH can complain of fatigue and, based on anecdotal reports from prior trials, the investigators are using magnetic resonance spectroscopy and blood/urine testing to assess muscle function. This illustrates the concept that the impact of burosumab on XLH may be far-reaching and affect multiple organ and tissue systems.

Cutaneous skeletal hypophosphatemia syndrome (CSHS) is a condition caused by somatic mutations in Ras and are characterized by excess FGF23 and skeletal dysplasias (25). Consequently it is a condition that may be amenable to burosumab therapy, which is currently being assessed by trial NCT03993821. As this is an early phase trial that aims to only recruit a single participant, it is important for other centers to consider testing CSHS patients.

Mechanistically, the effects of burosumab on both osteoblasts and osteoclasts are being measured in a pair of France-based trials (NCT04159675, NCT04184661) known as HYPO-BLASTE and HYPO-CLASTE. Bone cells will be harvested from XLH patients getting surgery for craniosynostosis and controls having surgery for idiopathic craniosynostosis who are also receiving burosumab and/or 1,25(OH)_2_D. These will be used to culture osteoblasts or osteoclasts. The behavior of these bone cells in culture will be compared to controls, which may include patients with idiopathic craniosynostosis.

Although data to date has not shown evidence for the development of cardiac calcification or nephrocalcinosis acutely or in the first year of treatment, it is unclear whether this may be a longer-term concern. Indeed, while the pediatric studies do not report hyperphosphatemia with burosumab treatment, 5.9% of adults in the multicenter trial developed hyperphosphatemia (15). The normalization of serum phosphate may have effects on calcium homeostasis for individuals with XLH, hence long-term monitoring will be critical as part of Phase 4 studies. Although the benefits of burosumab have been widely described, its impact on some of the other phenotypic features of XLH (e.g., dental abscess, craniosynostosis, Chiari malformation, arthropathy, enthesopathy, etc.) remain unanswered. The optimal maintenance dose in adults who have been treated with burosumab as a child also requires further study.

Finally, there is a long-term global, multicenter, longitudinal 10-year study (NCT03651505) to examine the changes in biomarkers, clinical assessments, and patient/caregiver-reported outcomes in a large XLH patient cohort both receiving burosumab and or conventional therapy. This should provide important safety and efficacy information as burosumab becomes a more established and common therapeutic intervention.

## Potential Risks of Burosumab Therapy

Overall the number of adverse events seen with burosumab therapy are believed to be more numerous than with conventional therapy (21), however the number of severe adverse events that have been linked to treatment remain rare. However, there are certain caveats linked to such a statement, and a need for further research in this space.

One of the major concerns associated with burosumab has been the potential for severe AEs associated with pathological tissue calcification. Based on the study by Imel et al. (10), transient hypercalcemia was rare and detailed monitoring was performed by cardiac CT and renal ultrasonograms. In this study, pre-existing nephrocalcinosis was not found to progress, and only one subject showed a notable increase in coronary artery calcification upon completion of dose escalation. Nevertheless, these complications need to continue to be monitored for by long-term research studies and followed in a longitudinal manner. Cardiac calcification has been noted with conventional therapy (phosphate and calcitriol) in rare instances (10). An important consideration is that many burosumab trials feature highly specific recruitment guidelines that could limit at-risk individuals. For example, in some clinical trials a low GFR score was noted as an exclusion criteria.

One notable AE that may be particularly important for pediatric care is the increased rate of dental abscess with burosumab treatment (31% burosumab vs. 6% placebo) (21). Low dentin mineralization and periodontitis are common in XLH, and it remains unclear whether this is due to patient variation or a specific relative risk of burosumab vs. conventional therapy.

Individuals with XLH can have a high rate of fractures and pseudofractures, and thus the impact of any intervention needs to be evaluated in this context. Some studies have suggested improvements in fracture/pseudofracture healing (15) and bone histology (17) with burosumab therapy. However, these studies also indicate a high baseline fracture and pseudofracture rate (47.1% in the burosumab treatment group, 57.3% in the placebo group) and notably a large proportion of fractures remained unhealed at the final time point (15). Thus, it is likely such orthopedic complications will still need to be carefully monitored and managed, even once burosumab therapy has been initiated, although further study will need to be undertaken to assess this.

## Conclusions

The development of burosumab as a therapeutic agent has been transformative for the treatment of XLH and is superior to conventional management of the condition in both adults and children. There have been multiple clinical reports from a range of sources that show consistent data supporting its continued use. Further clinical trials will be required to justify its use in other conditions that feature FGF23 dysregulation and follow up of existing patients on burosumab will be important to monitor for any potential long-term risk factors and adverse events. As burosumab therapy is being adopted worldwide as first-line treatment for XLH, replacing conventional phosphate/calcitriol therapy, collaborative international patient registries are necessary to enable ongoing surveillance of its long-term safety and efficacy.

## Author Contributions

All authors contributed equally to the writing a review of this manuscript.

## Conflict of Interest

CM has received research funding from Kyowa Kirin. The authors acknowledge that Kyowa Kirin had no role in design, data collection and analysis, decision to publish, or preparation of the manuscript. The reviewer EI declared a past co-authorship with all authors to the handling editor.
